# Participatory longitudinal qualitative interview study to understand Autistic gynaecological and obstetric health: the Autism from menstruation to menopause study protocol

**DOI:** 10.1136/bmjopen-2024-088343

**Published:** 2024-12-15

**Authors:** Aimee Grant, Gemma L Williams, Harriet Axbey, Abigail Wilkins, Ellen Firth, Hazel Lim, Helen Cave, Kathryn Williams, Kay Ribbons, Madeleine Sinfield, Monique Craine, Selena Caemawr, Willow Holloway, Amy Brown, Christina Nicolaidis, Helen Kara, Rebecca Ellis

**Affiliations:** 1 Swansea University, Swansea, UK; 2 Cardiff University, Cardiff, UK; 3 Portland State University, Portland, Oregon, USA; 4 We Research It Ltd, Uttoxeter, Staffordshire, UK

**Keywords:** QUALITATIVE RESEARCH, Community-Based Participatory Research, Disabled Persons, GYNAECOLOGY, Health Services Accessibility, OBSTETRICS

## Abstract

**Abstract:**

**Introduction:**

Autism is a lifelong minority neurotype present from birth. There is a dearth of credible evidence to suggest gender variation in Autism prevalence, despite historical under-diagnosis of women. Autistic people Assigned Female At Birth (AFAB) have worse physical and mental health compared with non-Autistic peers. To date, the reproductive health experiences of Autistic AFAB people have been under-investigated.

**Methods and analysis:**

This study aims to co-develop a quality improvement intervention to improve the reproductive health of Autistic people. The study uses Community Partnered Participatory Research (an approach similar to Community-Based Participatory Research), largely through a Community Council that co-governs the study. To understand reproductive health needs, a longitudinal qualitative investigation using creative methods will be undertaken with 100 Autistic AFAB people with 10 waves of data collection over 5 years (interview n=500–1000). Participants will be purposively selected to include harder-to-reach members of the Autistic community, including those who are non-speaking or semi-speaking, have a learning disability and those from marginalised ethnicities. Data will be analysed thematically with Community Council involvement. Intervention development will be undertaken from 2029 onwards.

**Ethics and dissemination:**

We are an Autistic-led team that adopts a social model of disability. However, this study raises ethical issues relating to sensitive topics and marginalised populations. Accordingly, we have robust procedures in place to assess capacity to ensure informed consent and to allow participants to take part without opting into data sharing. Ethical approval has been awarded by the Swansea University School of Health and Social Care Research Ethics Committee. We will publish our findings as open access articles in peer-reviewed journals.

**Strengths and limitations of this study**

- The study is co-governed by a Community Council of Autistic adults, and the lead researcher and academic research team are Autistic.
- The study uses qualitative longitudinal interviews over a 5-year period.
- We purposively sample those often under-represented in Autism research.
- The research is unlikely to be accessible to all Autistic people with learning disabilities or high support needs.
- Lessons from the study may not be transferrable to other contexts.

## Introduction

 Autism is a lifelong neurotype associated with differences in communication and sensory processing which can be disabling in a world designed for non-Autistic people. Autistic people (The language used in this paper - including the capitalisation of the A in Autism and Autistic which is a marker or culture, identity and community - is used with the best intentions, and we believe it to be best practice at the time of writing.^1^) have worse physical and mental health than non-Autistic peers and a shorter life expectancy.^2^ Estimates based on diagnoses of American children suggest that almost 3% of people are Autistic.^3^ However, women and others Assigned Female at Birth (AFAB) have been historically underdiagnosed, despite a dearth of credible evidence to suggest gender variation in Autism prevalence.^4^ The majority of Autism research funding is directed to research on children, and Autistic adults have been inadequately involved in Autism research which is about them.^5^ Furthermore, the lived experiences of AFAB Autistic people have been under-researched, with recent systematic reviews identifying relatively few papers.^6 7^ This is of particular importance because there is evidence to suggest that Autistic women have worse mental and physical health than their male counterparts. For example, Autistic women die by suicide four times more often than Autistic men^8^ and are more likely to suffer from chronic pain.^9^ This may be in part due to medical misogyny having an additional impact on Autistic AFAB people.^10^

In addition, gynaecological health (including menstruation, contraception and pelvic pain), obstetrics and menopause have been under-researched in the general population.^11^ It is well established that Autistic people experience bodily differences relating to hypermobility including Ehlers Danlos Syndrome,^12^ interoception (the ability to sense internal cues, such as hunger)^13^ and proprioception (the ability to sense where the body is in space).^14^ There is also some, although limited, evidence that Autistic AFAB people have different experiences to non-Autistic peers in relation to reproductive health. For example, feeling overwhelmed and reporting greater impacts of pain during menstrual periods,^15^ and experiencing increased sensory sensitivities during breastfeeding^16^ and menopause.^17^ When it comes to healthcare experiences related to reproductive health, Autistic people also report issues when interacting with healthcare environments.^18^ These are associated with adverse health outcomes.^19^ For this reason, there is an urgent need to better understand the reproductive health experiences of Autistic AFAB people.

## Methods and analysis

### Research questions

1. What are the reproductive health needs of Autistic AFAB people ?
2. How can the reproductive healthcare needs of Autistic AFAB people be better met within the UK National Health Service?

### Aims and objectives

Overarching aim: to co-develop a quality improvement intervention to improve reproductive health services provided to Autistic AFAB people.

Aim 1: Use a Community Partnered Participatory Research^20^ approach to do Autism research with Autistic people as equal partners throughout the project.

- *Objective 1.1*: Use a Community Partnered Participatory Research,^20^ akin to Community Based Participatory Research,^21^ approach throughout the entire project based on best practice for involving Autistic people in research.^5^
- *Objective 1.2*: Build Autistic community capacity in research skills through the creation of a fully accessible Autistic Research Community Council (ARCC) (n=12).
- *Objective 1.3*: Involve members of the ARCC as co-researchers in Aim 2 and Aim 3.
- *Objective* 1.4: Evaluate the collaboration between lay and academic researchers in the ARCC.
- *Objective* 1.5: Use social media to enhance Autistic community knowledge and understanding of research, following best practice guidance.^22^

Aim 2: Gain a comprehensive understanding of Autistic AFAB people’s reproductive health needs throughout the lifecourse, by co-producing, within a Critical Autism Studies paradigm, an in-depth examination of the gynaecological, obstetric and family-planning issues that affect them.

- *Objective 2.1*: Purposively recruit 100 Autistic AFAB people from four key points across the lifecourse.
- *Objective 2.2:* Co-produce ≤1000 (≥500) longitudinal qualitative interviews using participant diaries, visual and creative tasks and elicitation interviews (synchronous or asynchronous per participant choice) over a 5-year period.
- *Objective 2.3*: Undertake thematic data analysis with the ARCC.

Aim 3: Co-produce a quality improvement toolkit for Autistic AFAB people which aims to improve reproductive health and/or healthcare.

### Theoretical approach

The study will be situated within Critical Autism Studies^23^ and Feminist Disability Studies^24^ paradigms, both of which are politically situated with a focus on social and cultural structures that have the power to marginalise. This will be operationalised through the use of Community Partnered Participatory Research,^20^ an approach previously used within participatory Autism research.^25^

### Systematic reviews

We are undertaking three systematic reviews to situate the project, all of which have been prospectively registered:

1. Menstruation (PROSPERO registration: CRD42023399674; Ellis *et al*, under review).
2. Puberty (PROSPERO registration: CRD42023446750).
3. Menopause (PROSPERO registration ID: CRD42023450736).

### Patient and public involvement

We aim to include patient and public involvement (PPI) representatives as co-leaders of the study at every stage of the research. As such, the Autistic community has been involved in designing and co-governing the study from the outset.

#### Pre-application consultation

We undertook a community consultation in 2022 prior to applying for funding for this study. This consultation was undertaken via social media (Twitter, now known as X, and Facebook) and included two pre-existing Facebook groups with a focus on Autistic maternity, as this was the originally planned focus of the study. In this consultation, we received strong and clear feedback that other areas of reproductive health were essential to include in the study. Other feedback included the need to use gender-neutral language and identity first language relating to Autism (eg, ‘Autistic person’). In addition, two Autistic community leaders (KW and WH) were involved in discussions over several months to shape the research design, and they reviewed the full grant application. They were remunerated for their time via the Health and Care Research Wales Research Involvement Team. We have described the process of developing the grant application collaboratively in more detail elsewhere.^26^

#### Recruiting the research team

One of the Autistic community leaders (WH) was involved as an equal partner in recruiting the academic research staff for the project. This included reviewing applications (CVs, cover letters and a statement as to how applicants would support Autistic participants during data production) shortlisting, developing interview questions and interviewing applicants. She received an honorarium for her time.

#### Developing the community council

The two Autistic community leaders, KW and WH, involved in developing the application met with AG and decided on two further Autistic community leaders to bring into the Community Council. The purpose of this initial Council of four members was to accessibly design an expression of interest for eight further members to join the Council. They also undertook asset mapping to identify ways in which we could reach more marginalised Autistic people when advertising the role. Members drew on lived experience to increase accessibility in the project’s recruitment processes, in addition to what is recommended in the literature, through a series of seven 2-hour meetings. Additionally, 1:1 pre-meetings or post-meetings were available and were regularly taken up by two people. This allowed for additional processing time and for meeting documents to be read aloud where needed. The community leaders and academic researchers have reflected on this process, and the feeling of safety that was created in this space in an article we titled ‘building our own house’.^27^ Our community leaders did not include anybody with learning disabilities (We use the term ‘learning disabilities’ in this paper, as it is used in a UK context. In other countries, including the USA, the equivalent term is ‘intellectual disabilities’), and so an additional 3-hour 1:1 meeting was held with one Autistic woman with learning disabilities, who reviewed all recruitment materials to be used to recruit the additional Council members. We acknowledge that this involvement would not have identified all issues for all people with learning disabilities. All of those involved received an honorarium for their time.

The expression of interest for additional Council members was shared widely on social media and by a range of organisations that represent Autistic people or marginalised groups, with the hope of recruiting a Council that was representative of Autistic people. The organisations included: Autistic UK, All Wales Disability First, Disability Wales, Learning Disability Wales, Disability Sport Wales, the National Autism Team (for Wales) and Autistic led organisations Aubergine café and the Autistic Women’s Empowerment Project. We asked those interested in joining the Council to complete a short online survey to provide their demographics and why they would like to join. Alternatively, they were able to contact one of the academic researchers (RE) who could provide a Word document with the same questions or go through the questions with them over the phone or via video call. Around half of the applicants requested help with their application. We had anticipated that all members would be based in Wales, but we were unable to find a non-speaking Autistic person based in Wales, so we involved somebody from England to ensure this group was represented in our Council.

#### Involvement in systematic reviews

The four community leaders were involved in the thematic synthesis of literature relating to Autistic peoples’ experiences of menstruation; our paper relating to this is currently under review. We are undertaking two further systematic reviews, on puberty and menopause, in which we have included members of the Council with recent relevant experience.

#### Further shaping research design

In July 2023, an additional eight members joined the Community Council. Between July and December 2023, three 2-hour meetings were held, allowing for introductions, ethics training and discussions about research design. This involved piloting our planned activity for the first interview, a timeline-facilitated life history interview. The Council members identified that it was difficult to think back across the lifecourse and to put items in order. For this reason, we altered our pre-interview task to instead think about topics (eg, menstruation, puberty), rather than aiming to promote a linear approach. As before, additional 1:1 meetings (via Zoom or WhatsApp messenger) were offered. They were taken up by those who (a) wanted to review Council documents with support before the meeting, (b) wished to provide additional thoughts on a topic from the agenda but would find it difficult to do so in writing and (c) were unable to attend meetings. In November 2023, 1:1 cognitive interviews^28^ were held with 11 Council members who reviewed all participant-facing documents and provided feedback, which was integrated unless there were conflicting opinions between members.

A fourth Community Council meeting was held in December 2023 to discuss the project’s purposive sampling strategy. This resulted in a decision to proactively aim to recruit those with learning disabilities, those who are non-speaking or semi-speaking, from minority ethnicities and trans men. We also built on our list of organisations we would ask to facilitate recruitment to try to reach these more marginalised members of the Autistic community. Members of the Council also used their own networks and social media to share our study advertisement.

#### PPI outcomes

To date, Community Council members have been involved in: shaping the study’s research focus; analysis of the existing literature; research design; development of participant-facing documents and public engagement strategy. We anticipate they will continue to play a very active role in providing feedback on data collection, contributing to analysis and writing papers from our main research study. To date, one Council member has lost contact with the study, and it is possible that the format of the PPI activities did not meet her needs. Additional evaluation of the Community Council will be undertaken annually throughout the project, and we will further reflect on the views and experiences of Council members, our outcomes, things that went well and those that did not go so well, to make improvements.

### Sample selection

#### Sample size

Our study aims to include 100 AFAB Autistic people as participants. In designing this qualitative study, we did not undertake a sample size calculation, but considered that 25 participants per lifecourse group, and a total of 100 participants, would provide significant ‘information power’,^29^ and is on the large side for qualitative studies,^30^ before we consider the study’s longitudinal nature. Furthermore, our aim is to conduct ≤1000 qualitative interviews, and a minimum of 500, which is a very large qualitative data set.

#### Inclusion criteria

All participants will be required to meet the following criteria:

- Autistic (those self-identifying will undertake a self-assessment using the RAADS-14^31^).
- Have a womb or previously had a womb (ie, have had a hysterectomy).
- Live in the UK and expect to continue living in the UK for the next 5 years.
- Willing to share their experiences of reproductive health.
- Able to communicate in English with fluency (either written or spoken, including via the use of assistive technology).

We do not exclude those who have learning disabilities or who are non-speaking. To ensure representation across the lifecourse, participants also need to fit into one of these four lifecourse groups during recruitment:

- Aged 16–21.
- Aged 22–40 who have not given birth.
- Aged 22–40 who have given birth.
- Aged >16 years and in perimenopause, menopause or post-menopausal and experiencing menopause symptoms.

During the study, we anticipate movement between these groups.

#### Recruitment strategy

Our recruitment materials and participant-facing documents followed existing best-practice guidelines,^5^ and were created with our Community Council members, including those with learning disabilities. To ensure that our materials were accessible to a broad audience, they were available: on the study’s website (using drop-down menus to see the answer to each question); as a YouTube playlist, with a short video for each question and answer (https://www.youtube.com/watch?v=ncS1crOAtNo&list=PLv2nqZgRa-DeDS92kKJOCEoW98oRjkhg4), and as a Word document formatted to be accessible to screen reading software. Easy Read versions of documents were also available. Help with the recruitment process was available by contacting the academic researchers by email, phone or WhatsApp. This included the option of reviewing the participant information materials (including videos) together with a researcher, with the option to check understanding and respond to any questions after each segment. We acknowledge that our approach may not be accessible to all with high support needs and learning disabilities.

Adverts asking eligible individuals to complete an expression of interest were circulated via a broad range of organisations, including Autistic UK, Chinese Autism, Learning Disability Wales, Fair Treatment for the Women of Wales, National Neurodivergence Team (Wales) and the Autistic Women’s Empowerment Project. They were also widely shared via Facebook, Instagram, TikTok and X. Recruitment of participants began in January 2024, and we closed general recruitment on 28 March 2024, having received 1095 recorded responses. However, purposive recruitment may occur until we have consented 100 participants.

Received expressions of interest will be assessed by the academic researchers. Those who appear to not be genuine participants, such as originating outside of the UK, not answering any of the open text questions or providing answers that do not appear to be related to the study, will be removed due to concerns about fake participants seen recently in Autism research which offers incentives.^32^ Autistic people as a whole are an under-served group, however, within the Autistic community there are further intersectional groups who are rarely seen in research, including those with learning disabilities, non-speaking people and black and brown people. As such, our participants will be purposively selected, aiming for a diverse sample in relation to demographics including ethnicity, education level, geographical location and support needs. Those who have learning disabilities, are non-speaking, semi-speaking or who are primarily reliant on Augmentative and Alternative Communication to communicate will be proactively recruited. For example, we have already reopened recruitment for those with learning disabilities from April to May 2024. We also aim to include a range of genders, including trans men. Correspondingly, we will aim to limit the number of highly educated white people in our sample, as they are over-represented in Autism research.^33^

### Data production

We aim to use flexibility in data production to meet participants’ needs as far as resources allow, which is common practice when using creative research methods.^34^ This approach will maximise the accessibility of the study to a wide range of Autistic people, encompassing significant variation in communication (including those who are non-speaking) and sensory impairments.^35^ However, it is anticipated that all data collection will be remote. This was recommended in our pre-funding consultation with Autistic adults. It also has benefits in that it will reduce costs to the study and reduce fatigue in the (Autistic) research team.

To provide consent, participants will meet with their researcher. They can bring along a support person if they wish. If there is any concern that the person might not have the ‘mental capacity’ to take part, a Mental Capacity Act checklist will be used. The researcher will talk to the potential participant through either the standard or easy read participant information form answering any questions. The researcher will then review the consent form (standard or easy read) with the participant, one item at a time. All participants will be required to consent to take part in the research, including their interview being recorded. Participants also have the option to consent to:

- Receive a copy of the study’s results (with the ability to specify all outputs or only those in which their data are included in).
- Their direct quotations being used in (a) academic outputs and/or (b) social media and/or (c) teaching.
- Data sharing with other research teams.

Once participants have consented to take part in the study, they will undertake an accessibility assessment with their named researcher. This will include discussion of:

- Communication needs and preferences, including during interviews and between-interview communication.
- Interview procedure, including synchronicity (synchronous/asynchronous) and mode (text, voice, or video).
- Accommodations needed to take part in interviews, including reminders, changes to the interview procedure and the inclusion (or not) of breaks.
- Topics that they do not want to be included or may find difficult to discuss.
- Signs of distress including any agreed actions to indicate distress, helpful things for the researcher to do if they are distressed, and when, and providing reassurance that they can promptly end the interviews if needed.

#### Interviews

Over a 5-year period, each participant will take part in up to 10 interviews, including (a) an optional pre-interview task to ‘gather their thoughts’ followed by (b) an elicitation interview based on their pre-interview task content or the list of topics provided in the pre-interview information. The pre-interview task was developed in collaboration with the study’s Community Council (n=11). We piloted the use of a single timeline template within a Council meeting, and found that it did not meet everyone’s needs and a linear timeline could feel unhelpfully restrictive. As such, we developed a suite of eight templates, which were further reviewed in 1:1 meetings with an academic researcher and Council member. Each interview will last around an hour. Waves of data production will be roughly 6 months apart. Data production is anticipated to be completed in late 2029.

Participants will be provided with a four-page document which is a guide to the interview process. This includes: the style of the interviews (informal; aiming to meet all accessibility needs); topics that are of interest to the study (see box 1); information on ‘preparing your thoughts’ before the interview, and a range of blank templates and fictitious example templates which could be used to prepare their thoughts in advance of the interview (online supplemental appendix 1). Participants can also choose to gather their thoughts in other ways, such as keeping a diary (video, audio, typed, hand-written), collaging (using an online application or scrapbook), using everyday objects or anything else that better suits their needs and preferences. We are hoping that participants will choose to focus the interview on reproductive health topics of relevance to them, which we anticipate may change during the course of the 5-year longitudinal data production period.

Box 1 **Topics the study is hoping to collect information on**

In the interview, we would like you to share information about your reproductive health, and reproductive healthcare**.**

Depending on your personal situation, this means things like:

- Puberty.
- Periods (menstruation), including pain and how you’ve managed it, changes in mood, pre-menstrual syndrome and premenstrual dysphoric disorder.
- Symptoms or health conditions like heavy periods, endometriosis, fibroids, polycystic ovarian syndrome.
- Contraception and sexual health.
- Healthcare for anything to do with having a womb (‘uterus’), like smear tests, painful periods, accessing contraception, gender identity services and anything to do with hormones.
- Anything to do with pregnancy, including fertility treatment, pregnancy loss, birth, breastfeeding and chestfeeding.
- Perimenopause (also known as menopause or ‘going through the change’).

Participants can choose to share their creative outputs with the research team, or to keep them private. Any participants who do not gather their thoughts in advance of the interview will be shown the list of topics in box 1 in an unstructured way, in the hope of supporting participants to discuss the topics most salient to them, rather than the researcher guiding them using a semistructured topic guide. We anticipate using the same prompts in each of the ≤10 interviews, in order to produce further data on participants’ previous experiences while also being able to understand changes in reproductive health over time, and to produce data on recent healthcare experiences. We may also add additional topics or semistructured questions about topics that have arisen in previous interviews, which are of interest to the research team (academic and lay Community Council members).

Spoken interviews will be audiorecorded and transcribed verbatim, and any written content will be collected alongside this. Where the researcher is speaking and the participant typing, one integrated transcript will be prepared.

### Data analysis

Facilitated by NVivo (2020, R1), we will undertake thematic analysis using both deductive and inductive line-by-line coding, an approach well suited to interpretive inquiry.^36^ As analysis will be undertaken by a team of five researchers with an expansive data set, some deductive codes will be pre-agreed by the academic researchers, largely matching the topics of interest (box 1) that were developed with the Community Council. They will cover areas of reproductive health and healthcare experiences. The analysis will be for both semantic (surface level) as well as latent (underlying) understandings. Deductive coding of transcripts will be undertaken as a group, in order to ensure shared understandings of deductive codes, until all researchers are confident. Following this, the researcher who conducted the interview will be responsible for its coding wherever possible.

Following the deductive coding, reflexive thematic analysis^37^ will be applied to parts of the extremely large data set, for example, all material deductively coded as relating to maternity. The initial two stages of reflexive thematic analysis, familiarisation and coding, will be undertaken by one of the academic researchers and reviewed and discussed in weekly data analysis team meetings. Community Council members will be involved in four stages of reflexive thematic analysis: combining codes into themes, reviewing themes, defining themes and writing up. Council member’s involvement in analysis will occur through undertaking training, sharing written materials and undertaking meetings on a 1:1 or small group basis. We will report on our positionality in a reflexive way in empirical outputs. The academic researchers’ NVivo files will be regularly merged, so that all four lifecourse stages can be considered together.

We may use some quantification within our qualitative analysis, such as highlighting the frequency that a code is used, and the number of participants impacted, to give a relative idea of importance. This data will be presented to Community Council members, to identify areas for the in-depth inductive analyses to specifically focus on. We envisage creating smaller groups of around 3–5 lay members from our Community Council (n=11) to work on each major piece of analysis and the subsequent write-up of papers. This is to ensure that data analysis meetings remain manageable for facilitators, meet the accessibility needs of Community Council members and meet the Committee on Publication Ethics publication standards necessary to be an author on academic outputs.

In addition to this thematic analysis, we will create a narrative account of each of our 100 participants which will be added to following each wave of data production. This will initially be used to ensure the full research team is familiar with the key points shared by each participant, but may also be used in a formal narrative analysis,^38^ although ensuring the anonymity of participants in this longitudinal study will be of primary importance. For this reason, any narrative analysis will be undertaken by the academic researchers alone, due to the quantity of data available and the potential for participants to be recognised by Community Council members. We may or may not publish this analysis, depending on if it would be possible to do so without the risk of participants being identified.

## Ethics and dissemination

### Ethical issues

We are aware of a large range of ethical issues in our study. As such, we have undertaken significant reading and research team training on topics including trauma-informed research, ethics of choice, managing relationships, handling sensitive topics, leaving the field and researcher safety. In addition, prior to data collection with the relevant groups, training has included creating a comfortable and safe interview environment for those with learning disabilities^39 40^ and for trans men.^41^ We have also provided training for the Community Council on ethical aspects of research including ethical principles (using the British Sociological Association principles),^42^ and an exploration of ethics as they apply specifically to Autism research. The Community Council regularly considers ethical issues in our monthly meetings.

Potential participants will review a detailed participant information sheet which follows a question and answer format. It will be available as a Word document (for those using assistive software), in Easy Read format and as a series of videos with subtitles (one video per question). The participant information directs anybody with questions to contact the research team by email, telephone or WhatsApp. Those who are interested in participating will complete a short online survey to provide their demographic details, to facilitate purposive sampling. From those who complete the expression of interest, we will consent at least 100 participants to produce 100 interviews in wave 1. The consent and onboarding process is in-depth to ensure truly informed consent, including reviewing the participant information sheet together, undertaking an assessment of mental capacity if there are any concerns about capacity and completing consent using a consent form or audiorecorded script. Those who wish to opt in to data sharing will review further information and provide consent using a second consent form or script. Consent will be provided in writing or audiorecorded, per participants’ preferences. Potential participants are welcome to have a supporter present throughout. Consent to participate in the study and for data sharing (if relevant) will be revisited at each wave of data collection.

Gynaecological health symptoms are often under-treated, with patients facing many years of pain prior to diagnosis.^11^ As such, the data production process is likely to involve participants discussing areas of their health and healthcare that are sensitive topics and could be distressing.^43^ This is particularly so compared with the general population, as we know that Autistic people have worse health,^2^ and face barriers to accessing healthcare.^19^ Furthermore, Autistic people are more likely to have experiences of trauma.^44^ Feedback from our community consultation was that discussing these topics could be cathartic, as participants would feel listened to by researchers. In order to protect participants, we are following an ethics of choice approach, where participants are provided with the topics of interest, and tools to decide what they feel comfortable sharing with researchers prior to the interview date, and can also choose their mode of participation, such as turning off their camera, or typing in the chat,^35^ which can make communication about challenging topics easier for many Autistic people. Offering choice to participants is known to build trust around sensitive topics, and may also create an empirical, as well as an ethical, benefit.^45^ Furthermore, we are taking a trauma-informed approach to data production, including being mindful of the language in our participant-facing interview guide, and aiming to use empathetic listening, while knowing that discussing trauma rarely leads to re-traumatisation.^46^

Emotion work is known to be present in much qualitative research and can be striking in research where trauma is discussed or there are elements of shared history.^47^ As such, as well as ethical issues impacting on the participants, there is potential for our team of Autistic researchers to be impacted, particularly by hearing traumatic experiences which may have salience in our own lives. When discussing sensitive topics, researchers may experience unanticipated disclosures, and hear stories that are distressing or personally triggering, which can lead to exhaustion and burnout.^43 48^ To combat this, our close-knit academic research team will aim to debrief from each interview within one working day with another member of the academic research team. Furthermore, AG, the study lead, will be available to discuss any areas of concern, with the academic researchers encouraged to discuss mild concerns, as well as those that may trigger our safeguarding policy.^49^ Furthermore, researchers will use a research diary (also known as field notes^50^) to immediately ‘decompress’ after interviews, and we will hold a weekly team meeting, including a focus on data production issues or analysis depending on the phase of the study.^51^

Autistic people are a stigmatised and persecuted group. Organisations exist that describe Autism as a disease, and actively campaign to ‘cure’ our neurotype, for example, Autism Speaks only removed the word ‘cure’ from their website in 2016 and continues to be viewed with distrust by the Autistic community. It is quite possible that this research will uncover that Autistic participants struggled throughout their lives in ways that are additional to that of neurotypical peers (as reported in the research literature). Our approach, using a social model of disability, is that these difficulties show the need for greater accommodations.^52^ Viewed through a deficit-based medical model lens, however, this same data could be used as evidence that being Autistic is a tragedy that needs to be cured, or that Autistic foetuses should be aborted.^53^ In order to manage this within the funder’s guidance on data sharing, we are giving all participants the opportunity to take part in the study without sharing their data with other research teams. For those who are interested in data sharing, additional consent will be received and checked at each data collection wave.

The study has received ethical approval from the Swansea University School of Health and Social Care Research Ethics Committee (Approval number: 3202478217735; Approval date: 16 January 2024).

### Dissemination plan

We have already written two articles about our early PPI processes, in the grant application stage^26^ and in the initial development of the Community Council.^27^ We plan to publish the findings of our research using Open Access Publishing. Furthermore, we have set up a study website (https://www.autismmenstruationtomenopause.com/) and a range of social media accounts, to share our findings in a way that is accessible to the Autistic community. Should participants opt-in to data sharing, data will be stored in the UK Data Service archive.

### Toolkit development

A major element of Critical Autism Studies,^23^ Community Partnered Participatory Research approaches^20^ and trauma-informed research^46^ is to aim to improve society with research. As such, following the qualitative longitudinal research, we will work with the Community Council, Autistic people and other interested parties to create a toolkit. The contents of the toolkit will aim to meet the unmet needs relating to reproductive health expressed during interviews. To ensure maximum benefit to the entire Autistic community, we will ensure that under-represented groups, including those with learning disabilities and marginalised ethnicities, are well represented in these discussions. We will also ensure that those who may be involved in delivering services are part of toolkit development to maximise the likelihood of feasibility and acceptability in practice.^54^ We will use separate groups (to foster accessibility) where required. The aim of this will be to improve Autistic people’s gynaecological and obstetric health and/or healthcare. We anticipate undertaking this element of the study in 2029–2030.

## supplementary material

10.1136/bmjopen-2024-088343 online supplemental file 1
