# Supplementary figures and images for: Participatory longitudinal qualitative interview study to understand Autistic gynaecological and obstetric health: the Autism from menstruation to menopause study protocol
Source: BMJ Open. 2024 Dec 15;14(12):e088343. doi: 10.1136/bmjopen-2024-088343 (PMC11647321; doi:10.1136/bmjopen-2024-088343)

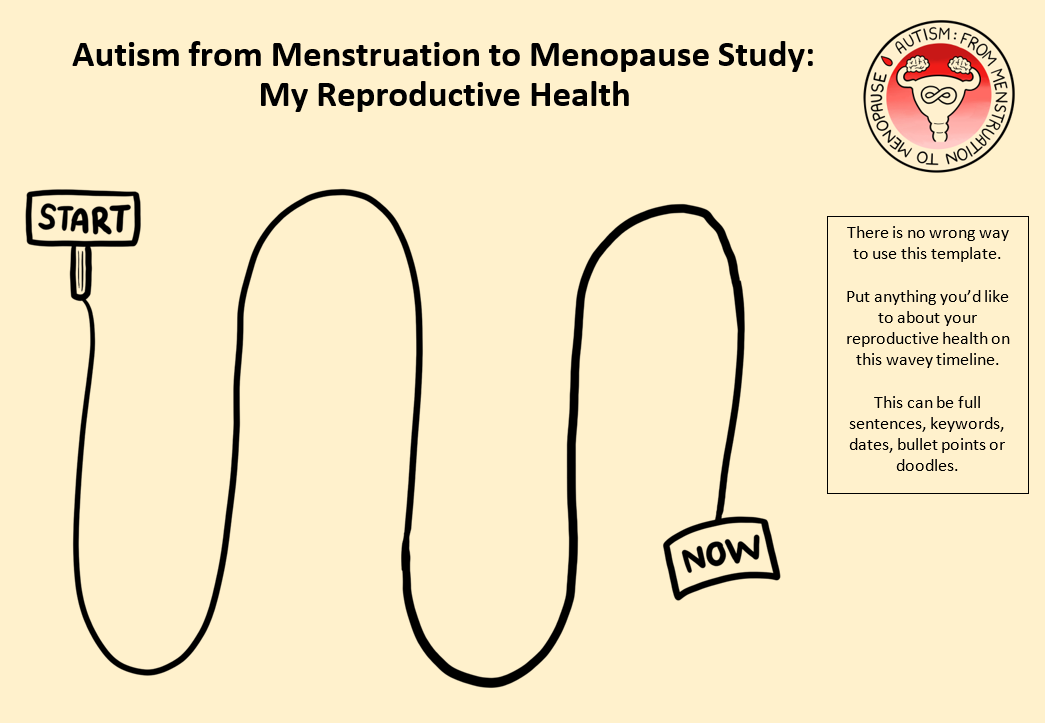


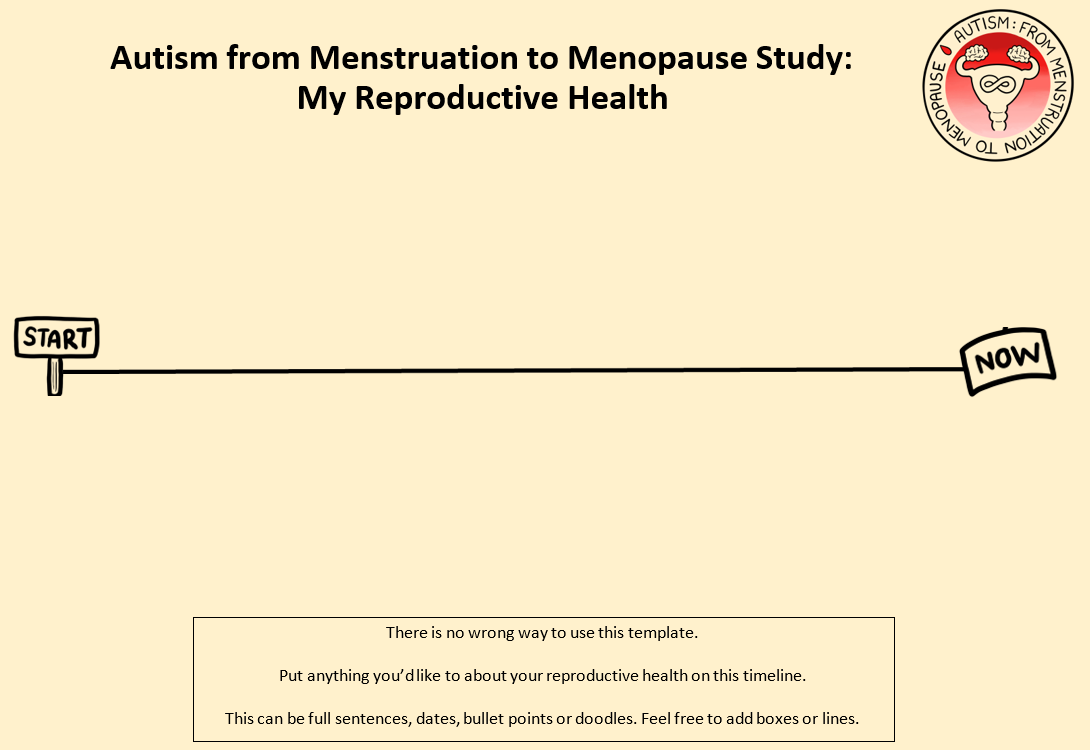


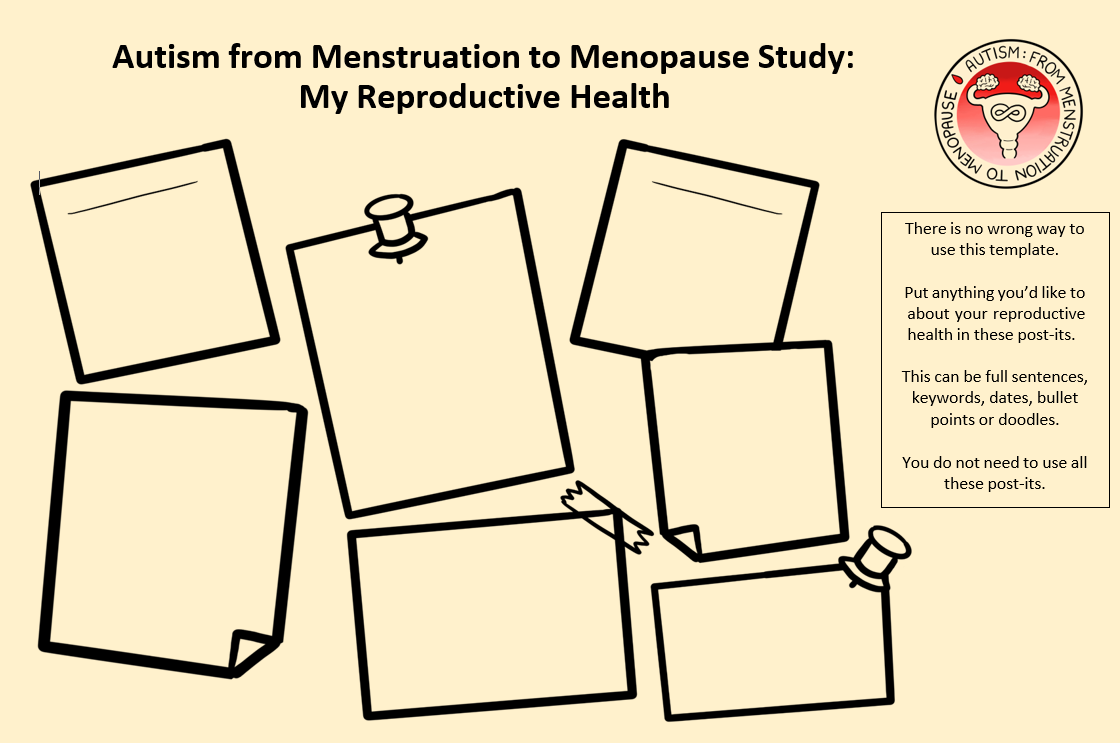

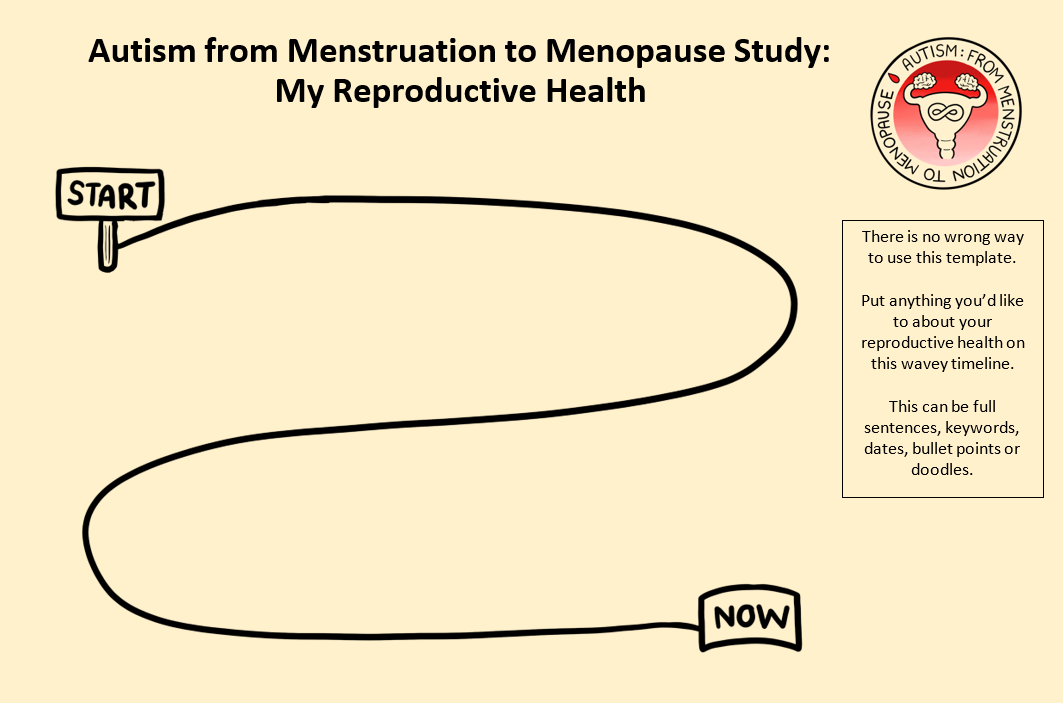


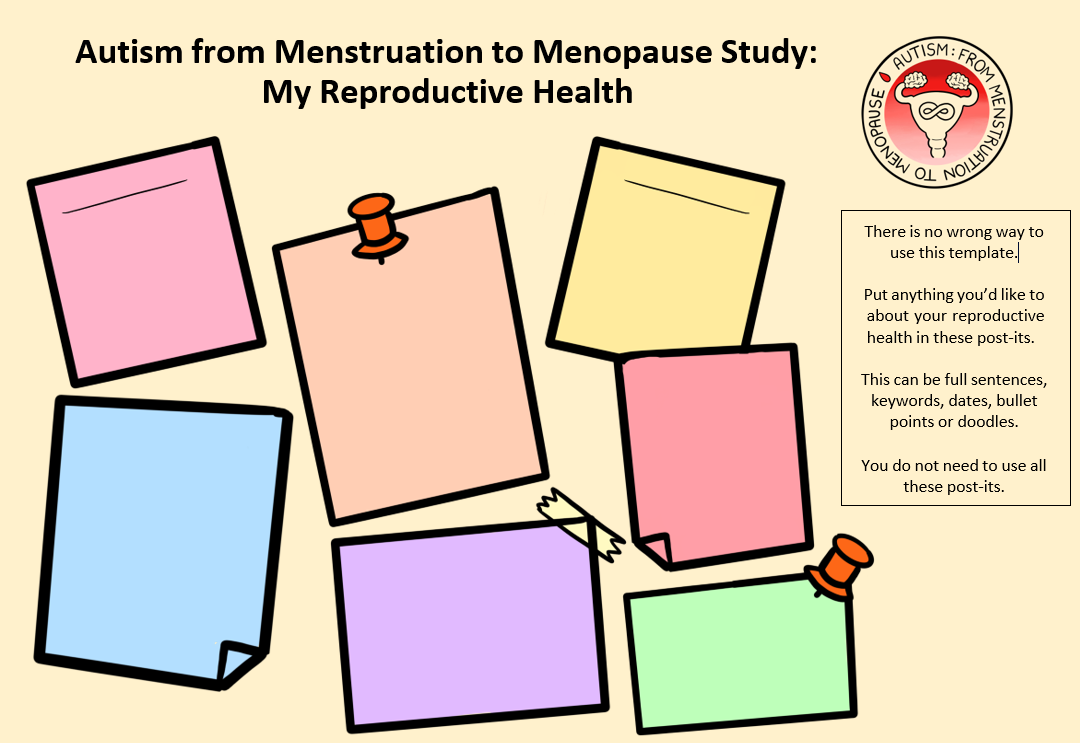

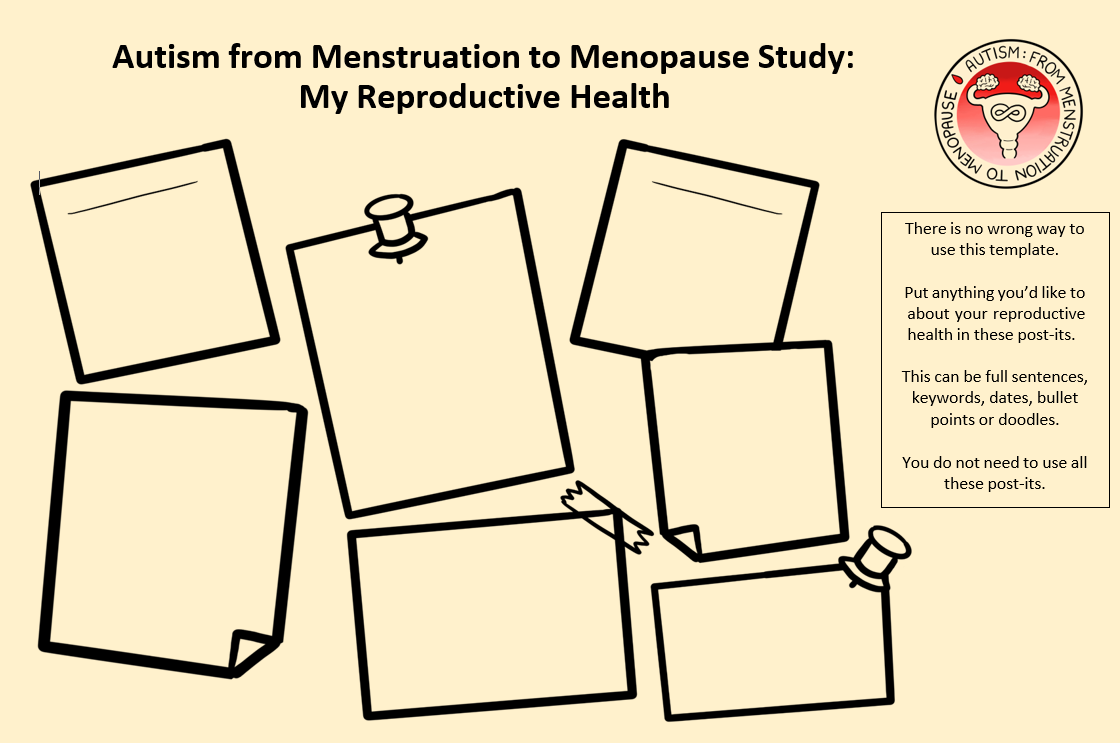


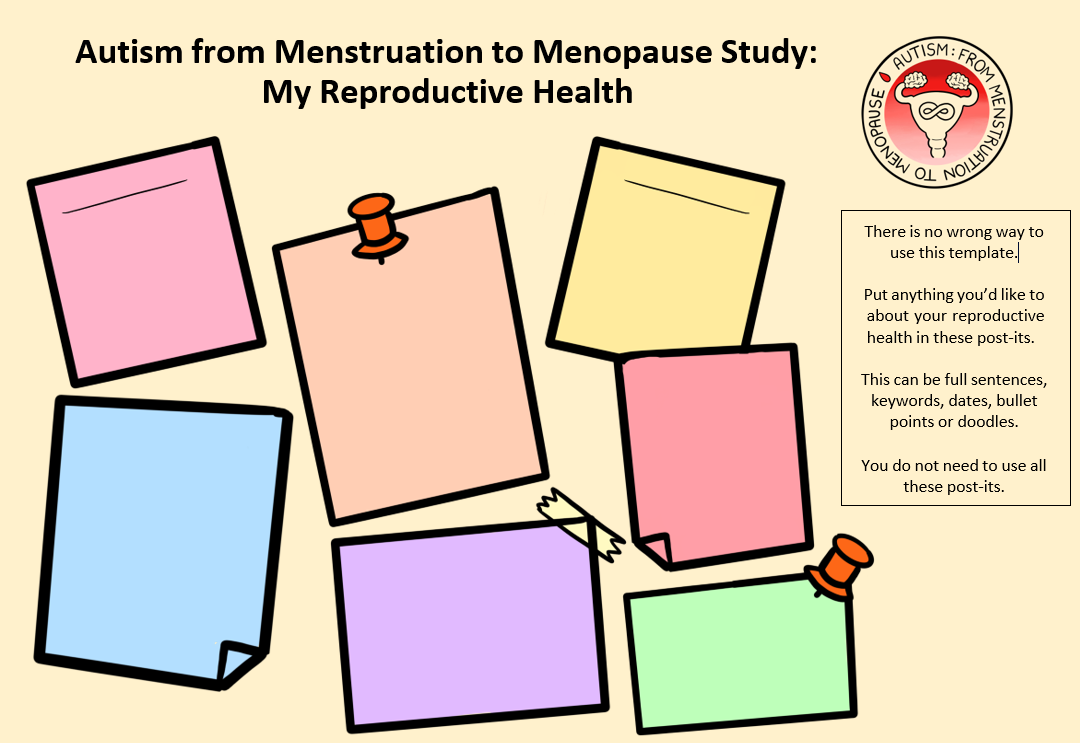


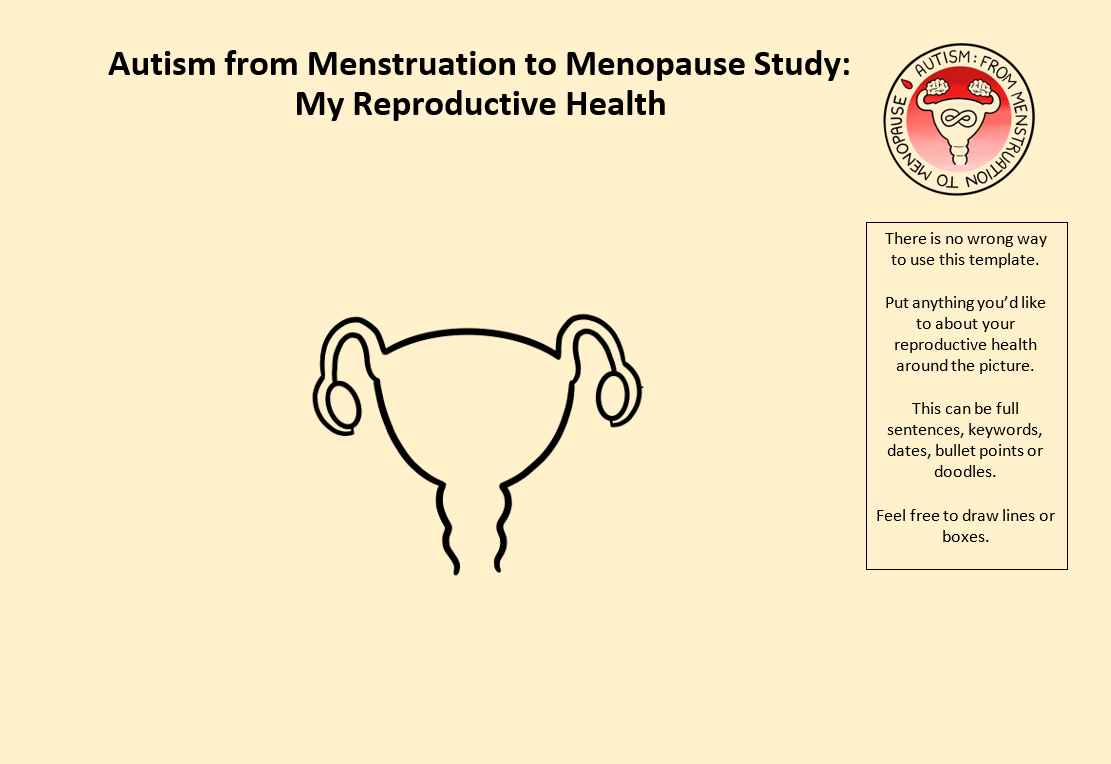


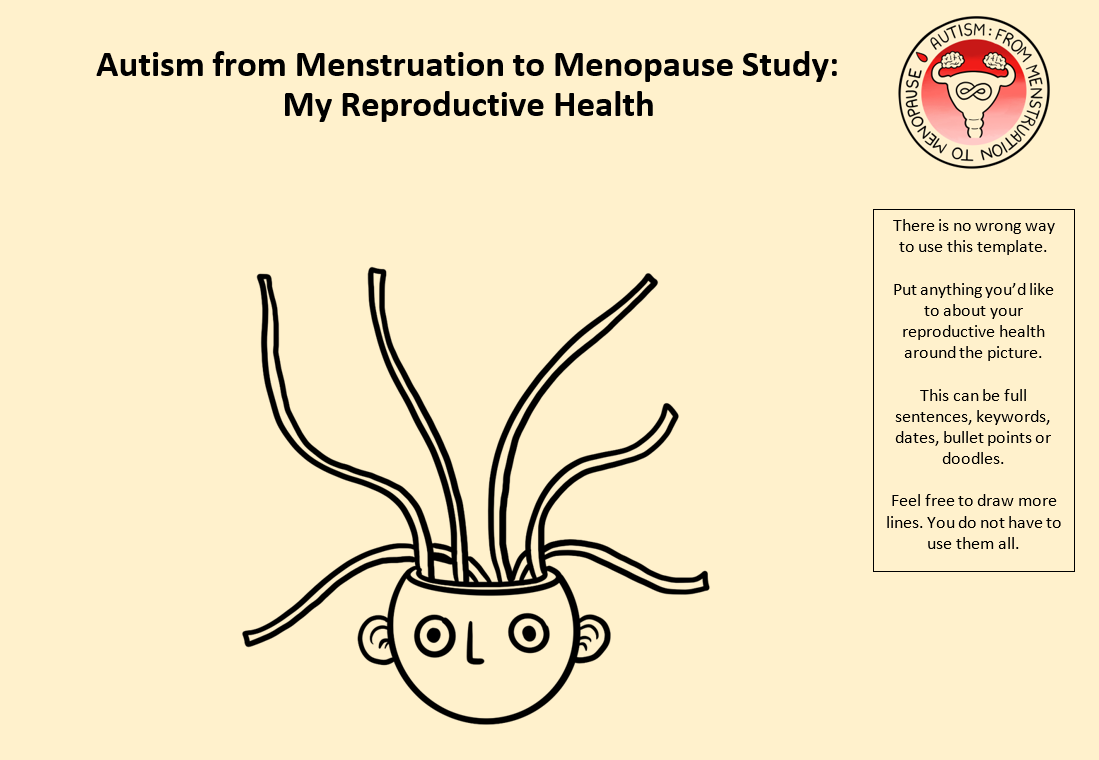


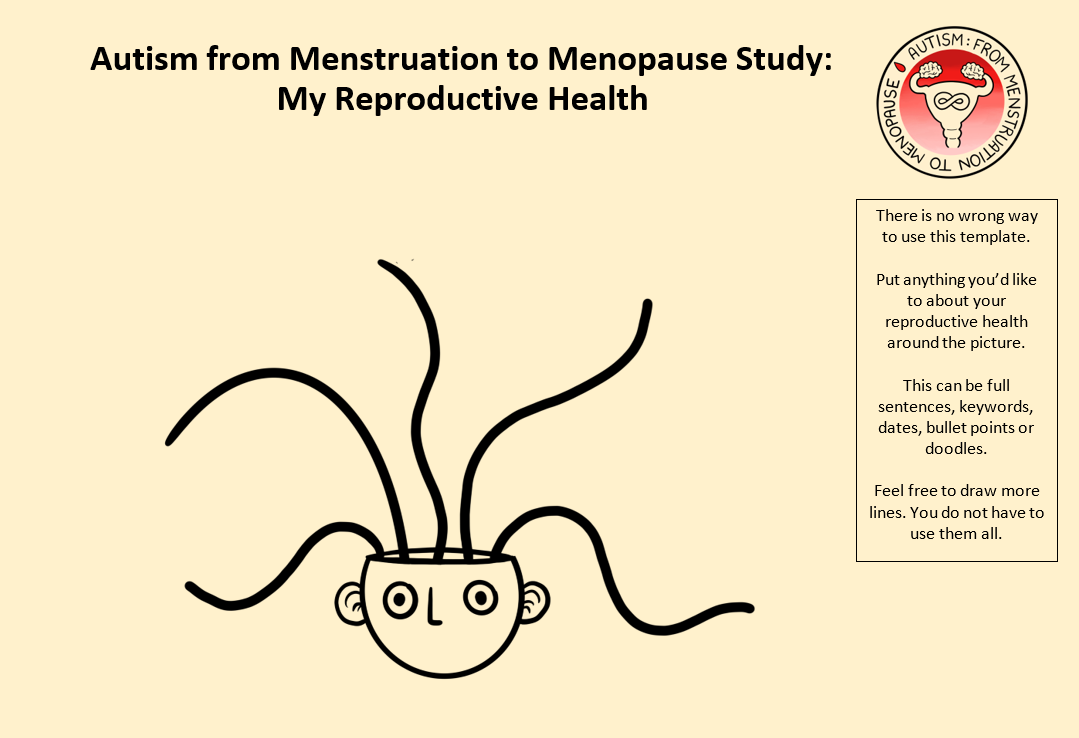

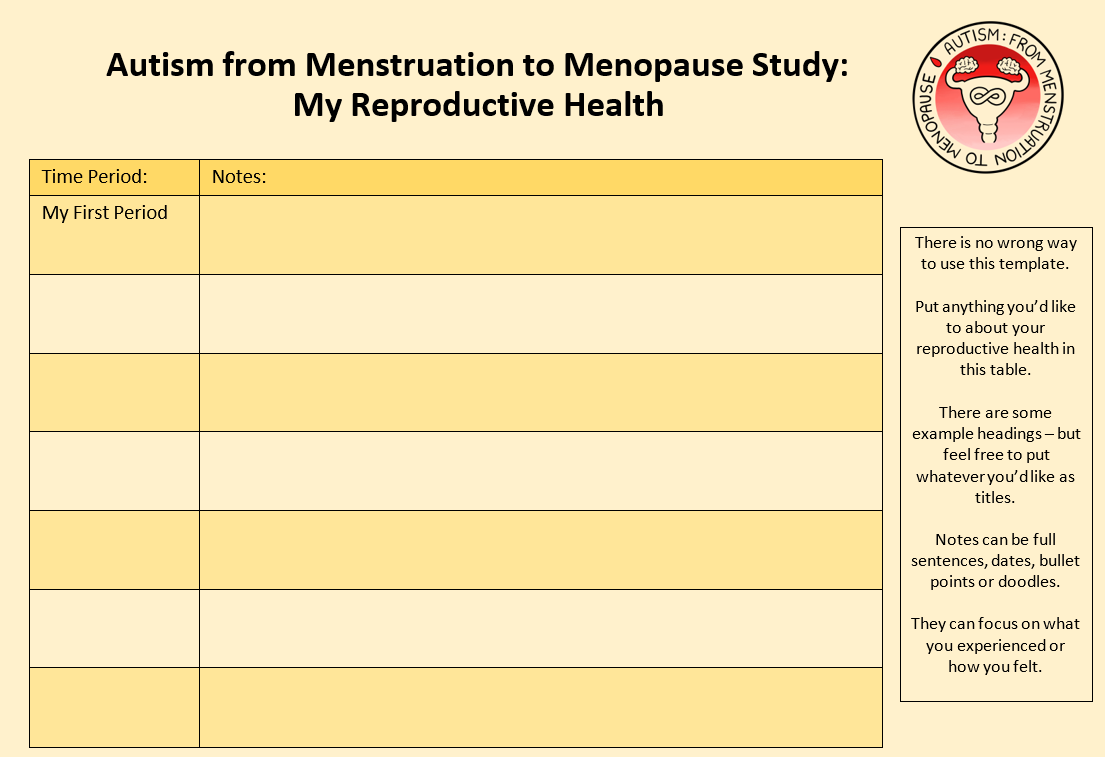

Supplement: online supplemental file 1 [file bmjopen-14-12-s001.docx]
