# Supplementary material for: Participatory longitudinal qualitative interview study to understand Autistic gynaecological and obstetric health: the Autism from menstruation to menopause study protocol
Source: BMJ Open. 2024 Dec 15;14(12):e088343. doi: 10.1136/bmjopen-2024-088343 (PMC11647321; doi:10.1136/bmjopen-2024-088343)
Supplement: Supplementary file 2 [file bmjopen-2024-088343.reviewer_comments.pdf]

PEER REVIEW HISTORY

BMJ Open publishes all reviews undertaken for accepted manuscripts. Reviewers are asked to complete a checklist review form and are provided with free text boxes to elaborate on their assessment. These free text comments are reproduced below.

ARTICLE DETAILS

Title (Provisional)

A participatory longitudinal qualitative interview study to understand Autistic gynaecological and obstetric health: the Autism from Menstruation to Menopause study protocol

Authors

Grant, Aimee; Williams, Gemma L; Axbey, Harriet; Wilkins, Abigail; Firth, Ellen; Lim, Hazel; Cave, Helen; Williams, Kathryn; Ribbons, Kay; Sinfield, Madeleine; Craine, Monique; Caemawr, Selena; Holloway, Willow; Brown, Amy; Nicolaidis, Christina; Kara, Helen; Ellis, Rebecca

---

VERSION 1 - REVIEW

**Reviewer**	**1**
**Name**	**Lunsky, Yona**
**Affiliation**	**Centre for Addiction and Mental Health, Underserved Populations Program**
**Date**	**31-Jul-2024**
**COI**	**I have no competing interests to declare.**

I so enjoyed reading this protocol. The authors have taken extensive care to ensure that research participants are safe and informed. They use many best practices to engage with and recruit participants, as well as to work with their Community Council. When they are limited (for example with involving people with intellectual disabilities (or learning disabilities as referred to in the UK), they acknowledge this and do what they can.

It is a unique study in that the lead researcher and the entire academic research team identify as autistic. There are not that many larger programs of research (this is estimated to last until 2030 with multiple data points for 100 participants) made up only of autistic people as there could be, and I am not familiar with many protocol papers such as this..

The authors have also taken great care to be sensitive to issues of gender diversity, and the difference between sex and gender. They speak about women and other people with a womb. Given the degree of gender diversity in the autistic community, this is especially important.

The study was built out of extensive community engagement and the final questions being asked are a result of that, and broader than the original study goal related to perinatal care.

Recognizing that it is difficult to change the protocol now, I have just a few comments or points of clarification. I am eager to learn more from this team about their study findings, and lessons learned through the way they are carrying out their research.

Community council and patient engagement

I appreciated many of the thoughtful details in this study and can see others citing this work not just for the content but also processes The only area where I had some concern was about making it inclusive of people with intellectual disabilities.(If authors choose to use the term learning disabilities, they should also indicate that this is also referred to as intellectual disabilities. Outside of the UK learning disability has a different meaning). It may be that meeting with 1 person for 3 hours would not be sufficient. Different individuals would have different learning preferences and ways of understanding materials. I do appreciate the acknowledgment of this limitation. It may be that for people who are both autistic and have ID, they will just pick certain topics to talk about (that is what is nice about the methods). I think the consent process may also need to be further simplified for this group. Authors may also want to mention the support they may receive from another person to comfortably participate in the study. Thinking forward, if there is a group of individuals with learning disability who participate in the study, then some consideration of how to translate findings for them would be important and I would suggest working with a group of advisors with learning disabilities for this work.

Recruitment

I think it will be helpful in the final protocol to hyperlink the various efforts from the website including the videos. I appreciate that watching the videos can help people who cannot or would prefer not to read, but simplifying the process and the language may also be helpful. It may be worthwhile to recognize that only a subgroup of people with intellectual disabilities would be able to participate. In addition, if people are invited to review the videos together with someone from the study, and pause after each segment, that would be useful to describe.

Fake participants

I appreciated the comments about fake participants. This is a big issue in online recruitment and can be particularly complicated in autism research where you want to make things low barrier and accessible but also ensure that everyone in the study is autistic. It would be helpful to indicate how the researchers would know/suspect people were fake besides looking at where they came from.

I really appreciated the details and accommodations around the interview process. These are definitely best practices and was so nice to read about.

I wanted to check if the team piloted the interview along with the preparing thoughts sheets. (I really liked the idea of the preparing thoughts preparatory work).

Analysis

There will be a lot of data for this study, as authors acknowledge. I liked the outline distributing the data for analysis, so that council members are not overwhelmed but still involved.

Ethics and dissemination

I appreciated considerations to being trauma informed not only with research participants but also with researchers. In debriefing after each interview, perhaps authors could indicate who the interviewers debrief with (the PI? a couple of team members?) I think the tensions around what research is done, and how it is funded and carried out, and what happens at the point of knowledge translation, and the impact this has on autistic people, families and the public is important to include in this section. I think this goes beyond specific organizations or studies so I am not sure examples of studies or organizations are required but the authors and council may have felt strongly that the specific examples be noted . I really do appreciate the tensions between open science and choice for this population with fears about how a person's data can be used, and I liked the decision to allow the cohort to make ongoing decisions at each wave of data about data sharing.

In summary, this is a very ambitious study which should lead to a richer understanding of critical issues, and also provide valuable insights into co-produced health research with the autistic population. It will take some time to complete, so the protocol paper outlining how the work is being done and why it is being done in this way is very helpful and can inform practice on these specific health issues as well as participatory research methods with autistic people.

**Reviewer**	**2**
**Name**	**Farmahini Farahani, Maryam**
**Affiliation**	**Islamic Azad University, nursing and midwifery**
**Date**	**04-Aug-2024**
**COI**	**I have not any competing interest.**

Dear author

The present study is very valuable and important. One of the positive points of this study is the high number of participants and the research team.

Some parts of the article were incomprehensible to me, so for the sake of clarity, please reply to my comments.

1-You mentioned in the study method “Recruitment strategy” that participants who were unable to speak or had learning disabilities were also included in the study. But you did not mention this group in the inclusion criteria (page 9). please explain more.

2- In the section “data collection” , this phrase: “That said, in order to meet the needs of the (currently entirely Autistic) research team and reduce costs to the study, it is anticipated that all data collection will be remote” is incomprehensible to me. please explain more.

3- Considering that one of the aims of your study is to examine reproductive health from puberty to menopause, why is the age range of the subjects of your study up to the age of 40?

4- Please explain, how you interviewed participants who were unable to speak or had learning disabilities?

5- Why did you not mention the topics you chose for the interview, breast examination and breast cancer screening?

6- Please re-edit the text of the article so that it is conceptually more readable and fluent.

VERSION 1 - AUTHOR RESPONSE

Reviewer 1:

- Participants in the non-mother group (group 2) could get pregnant and become part of group 3.
  - *Noted below the inclusion criteria that we anticipate movement between the life-course groups during the study.*
- How can I ensure the inclusion of Autistic people with high support needs/learning disabilities?
  - *We have noted throughout the manuscript ways that we have aimed to make the study more inclusive, but also that we may not be able to create a fully accessible study.*
- There is limited detail on the analysis process and how the Community Council will be included.
  - *I have added more information on exactly which parts of the thematic analysis the council will be involved in.*

- Ensure a thorough evaluation of the Council is conducted and shared
    - *This was already noted in 'PPI Outcomes' section.*

Reviewer 2:

- Research questions not included
    - *These were included in the grant application and have been added to the manuscript prior to the study's aims.*
- Interviews will not be standardised
    - *We believe that this is a benefit to the study, which will allow for participants to share the issues that are most important to them. We have added this sentence "We are hoping that participants will choose to focus the interview on reproductive health topics of relevance to them, which we anticipate may change during the course of the five-year longitudinal data production period."*
- What does the toolkit entail and which stakeholders will you include in developing it?
    - *Additional information on the toolkit has been added:*

The contents of the toolkit will aim to meet the unmet needs expressed during interviews. To ensure maximum benefit to the entire Autistic community, we will ensure that under-represented groups, including those with learning disabilities and marginalised ethnicities, are well represented in these discussions. We will also ensure that those who may be involved in delivering services are part of toolkit development to maximise the likelihood of feasibility and acceptability on the group. We will use separate groups (to foster accessibility) where required.

Reviewer 3

- Need to demonstrate a wider appreciation of the literature to properly situate the project
    - *We are undertaking three prospectively registered systematic reviews and have noted this below the 'theoretical approach' section.*
- What model of participatory research/coproduction will be used?
    - *We have noted our use of Critical Autism Studies, Feminist Disability Studies and CPPR.*
- Sensitive research topic – potential for an "intrusive threat" to participants

- *We have addressed this through our approach of allowing participants to guide the interview topics, and note this in the ethics section.*

- Ethics – the research could further stigmatise /marginalise the population
  - *We have noted this when discussing data sharing in the ethics section.*
- Creative methods – who will take the pictures participants respond to?
  - *We have noted that participants will be in charge of all creative methods used, and they will decide whether or not to share their creative outputs with their researcher.*
- 125 (should be 100) participants and 1000 interviews feels too ambitious – would I be able to find enough participants?
  - *We were able to identify over 1,000 people who wanted to take part in the study within two months. This has been noted.*
- Will self-identifying Autistic people be included?
  - *This is included in the inclusion criteria.*
- How will Autistic people be involved in the analysis?
  - *This is noted in the data analysis section.*
- Lack of researcher reflexivity noted re: analysis
  - *I have added a sentence that notes: "We will report on our positionality in a reflexive way in empirical outputs."*
- Should there be preliminary mapping work of services with health professionals, prior to intervention development?
  - *It is not possible for this work to be undertaken within this study, but it could be part of a follow-on study. As such, this has not been noted in this protocol paper.*
- How will health professionals be involved in intervention co-development?
  - *I have added a citation to a methods paper based on how to co-develop an intervention with NHS staff.*

Reviewer: 1
Dr. Yona Lunsky, Centre for Addiction and Mental Health, University of Toronto

I so enjoyed reading this protocol. The authors have taken extensive care to ensure that research participants are safe and informed. They use many best practices to engage with and recruit participants, as well as to work with their Community Council. When they are limited (for example with involving people with intellectual disabilities (or

learning disabilities as referred to in the UK), they acknowledge this and do what they can.

It is a unique study in that the lead researcher and the entire academic research team identify as autistic. There are not that many larger programs of research (this is estimated to last until 2030 with multiple data points for 100 participants) made up only of autistic people as there could be, and I am not familiar with many protocol papers such as this..

The authors have also taken great care to be sensitive to issues of gender diversity, and the difference between sex and gender. They speak about women and other people with a womb. Given the degree of gender diversity in the autistic community, this is especially important.

The study was built out of extensive community engagement and the final questions being asked are a result of that, and broader than the original study goal related to perinatal care.

Thank you for your positive comments.

Recognizing that it is difficult to change the protocol now, I have just a few comments or points of clarification. I am eager to learn more from this team about their study findings, and lessons learned through the way they are carrying out their research.

Thank you – we will be sharing updates on our study website – www.autismmenstruationtomenopause.com and the social media accounts shared on our website.

Community council and patient engagement

I appreciated many of the thoughtful details in this study and can see others citing this work not just for the content but also processes.

Thank you, this is appreciated.

The only area where I had some concern was about making it inclusive of people with intellectual disabilities. It may be that meeting with 1 person for 3 hours would not be sufficient. Different individuals would have different learning preferences and ways of understanding materials. I do appreciate the acknowledgment of this limitation.

We have added an extra sentence to the section where we describe this individual's involvement to say: "We acknowledge that this involvement would not have identified all issues for all people with learning disabilities."

(If authors choose to use the term learning disabilities, they should also indicate that this is also referred to as intellectual disabilities. Outside of the UK learning disability has a different meaning).

> *We have added "(also known as intellectual disabilities)" and a footnote the first time we use 'learning disabilities'.*

It may be that for people who are both autistic and have ID, they will just pick certain topics to talk about (that is what is nice about the methods).

> *Thank you, this was definitely our intention and hope for the study.*

I think the consent process may also need to be further simplified for this group. Authors may also want to mention the support they may receive from another person to comfortably participate in the study.

> *Thank you for this important comment – I realise on reflection that we have not described our consent process. This has been added to the "data production" section of the manuscript.*

Thinking forward, if there is a group of individuals with learning disability who participate in the study, then some consideration of how to translate findings for them would be important and I would suggest working with a group of advisors with learning disabilities for this work.

> *We absolutely agree with you. To ensure we meet the needs of our Community Council members who will be authors on academic outputs, all papers will have a lay summary developed whilst writing. When sharing our findings with participants we will use easy read and plain English.*
>
> *We will also invite a broad range of stakeholders (likely in separate meetings to ensure accessibility), when we develop the toolkit in years 7 and 8. To make this clearer, I have added this sentence:* "To ensure maximum benefit to the entire Autistic community, we will ensure that under-represented groups, including those with learning disabilities and marginalised ethnicities, are well represented in these discussions, through the use of separate groups (to foster accessibility) if required."

Recruitment

I think it will be helpful in the final protocol to hyperlink the various efforts from the website including the videos. I appreciate that watching the videos can help people who cannot or would prefer not to read, but simplifying the process and the language may also be helpful. It may be worthwhile to recognize that only a subgroup of people with intellectual disabilities would be able to participate. In addition, if people are invited to review the videos together with someone from the study, and pause after each segment, that would be useful to describe.

Thank you for this point. We have added a link to the video playlist and a note that people could review the participant information (in any form) with a researcher. As there is not a limitations section in the BMJ Open protocol paper format, we have also added a note that we acknowledge that our methods will not be accessible to all with high support needs and learning disabilities.

Fake participants

I appreciated the comments about fake participants. This is a big issue in online recruitment and can be particularly complicated in autism research where you want to make things low barrier and accessible but also ensure that everyone in the study is autistic. It would be helpful to indicate how the researchers would know/suspect people were fake besides looking at where they came from.

> *In addition to the geographic location, we have also had some responses that don't seem relevant to the study (or being Autistic at all), which we suspect may be AI generated. We very much used the cited article to guide our sifting:* Pellicano E, Adams D, Crane L, *et al.* Letter to the Editor: A possible threat to data integrity for online qualitative autism research. *Autism*. 2023;136236132311745.

I really appreciated the details and accommodations around the interview process. These are definitely best practices and was so nice to read about.

> *Thank you.*

I wanted to check if the team piloted the interview along with the preparing thoughts sheets. (I really liked the idea of the preparing thoughts preparatory work).

> *We didn't completely pilot the interview process, but we did spend a Council meeting trialling the use of a single timeline template. We found that it was difficult for some members to use in practice, due to the linear nature. As such, we used the feedback to create a group of seven templates, which we then received further feedback on, resulting in one additional template being added. We have updated the interviews section of the manuscript to report on this.*

Analysis

There will be a lot of data for this study, as authors acknowledge. I liked the outline distributing the data for analysis, so that Council members are not overwhelmed but still involved.

> *Thank you.*

Ethics and dissemination
I appreciated considerations to being trauma informed not only with research participants but also with researchers. In debriefing after each interview, perhaps authors could indicate who the interviewers debrief with (the PI? a couple of team members?)

> *I have updated this section to note that the debrief is with another one of the academic researchers, but that in addition, I should be contacted if there are any concerns.*

I think the tensions around what research is done, and how it is funded and carried out, and what happens at the point of knowledge translation, and the impact this has on autistic people, families and the public is important to include in this section. I think this goes beyond specific organizations or studies so I am not sure examples of studies or organizations are required but the authors and council may have felt strongly that the specific examples be noted . I really do appreciate the tensions between open science and choice for this population with fears about how a person's data can be used, and I liked the decision to allow the cohort to make ongoing decisions at each wave of data about data sharing.

> Thank you for this point. I have removed the name of the study that was previously included in our Community Council ethics training session. Thank you for your support for our data sharing policy, which errs on the side of participant choice and caution.

In summary, this is a very ambitious study which should lead to a richer understanding of critical issues, and also provide valuable insights into co-produced health research with the autistic population. It will take some time to complete, so the protocol paper outlining how the work is being done and why it is being done in this way is very helpful and can inform practice on these specific health issues as well as participatory research methods with autistic people.

Thank you for your detailed and supportive comments – they are very much appreciated.

Reviewer: 2
Dr. Maryam Farmahini Farahani, Islamic Azad University

Dear author
The present study is very valuable and important. One of the positive points of this study is the high number of participants and the research team.

> *Thank you.*

Some parts of the article were incomprehensible to me, so for the sake of clarity, please reply to my comments.

1-You mentioned in the study method "Recruitment strategy" that participants who were unable to speak or had learning disabilities were also included in the study. But you did not mention this group in the inclusion criteria (page 9). please explain more.

Thank you for this point. Having learning disabilities or being non-speaking isn't mentioned in our inclusion criteria, because we do not exclude people who do not have learning disabilities or who are speaking. To make it very clear that they are included, we have added a sentence below our inclusion criteria that states: "We do not exclude those who have learning disabilities or who are non-speaking."

2- In the section "data collection" , this phrase: "That said, in order to meet the needs of the (currently entirely Autistic) research team and reduce costs to the study, it is anticipated that all data collection will be remote" is incomprehensible to me. please explain more.

Apologies for this unclear sentence. It has now been updated to:

However, it is anticipated that all data collection will be remote. This was recommended in our pre-funding consultation with Autistic adults. It also has benefits in that it will reduce costs to the study and reduce fatigue in the (Autistic) research team.

3- Considering that one of the aims of your study is to examine reproductive health from puberty to menopause, why is the age range of the subjects of your study up to the age of 40?

Apologies – I think you have misread the relevant section as our fourth life-course group goes beyond the age of 40 (any age over 16 experiencing menopausal symptoms). In our inclusion criteria section we previously noted: "Aged over 16 years and in perimenopause, menopause or post-menopausal and experiencing menopause symptoms." To make it clearer that participants only need to fit into one of the four life course groups, the word "one" has now been bolded, and we have changed the 'over' to '>16 years'. "However, to ensure representation across the life course, participants also need to fit into **one** of these four life-course groups... Aged >16 years and in perimenopause, menopause or post-menopausal and experiencing menopause symptoms"

4- Please explain, how you interviewed participants who were unable to speak or had learning disabilities?

Participants are able to select whichever mode of interview best meets their needs. This means that those who are non-speaking can type their answers, either in real time during a video interview (eg: using zoom) or asynchronously (eg: by email or WhatsApp). We have added to the data production section to make this clearer:

"We aim to use flexibility in data production to meet participants' needs as far as resources allow, which is common practice within creative research methods.[34] This approach will maximise the accessibility of the study to a wide range of Autistic people, encompassing significant variation in communication (including those who are non-speaking)."

There is a large body of research which involves interviewing those with learning disabilities. We have followed best practice on this study, and note in the 'ethical issues' section: "In addition, prior to data collection with the relevant groups, training has included creating a comfortable and safe interview environment for those with learning disabilities [38,39]".

5- Why did you not mention the topics you chose for the interview, breast examination and breast cancer screening?

The key topics (displayed in Box 1) were developed in collaboration with the study's community council. We didn't want to provide an exhaustive list, as Council members thought that the list length could be overwhelming to participants if it was too long. However, participants are free to discuss any topics related to reproductive health, including breast examination and breast cancer screening. To help guide the reader, I have added the sentence: "We are hoping that participants will choose to focus the interview on reproductive health topics of relevance to them, which we anticipate may change during the course of the five-year longitudinal data production period."

6- Please re-edit the text of the article so that it is conceptually more readable and fluent.

The manuscript was professionally proofread prior to submission, but we have sent it to be proof read by a different proof reader.

VERSION 2 - REVIEW

Reviewer 1

Name Lunsky, Yona

Affiliation Centre for Addiction and Mental Health, Underserved Populations Program

Date 17-Nov-2024

COI

I appreciate responses to reviewer concerns.
